# Impact of educational agents on student’s learning outcomes: a meta-analysis

**DOI:** 10.3389/fpsyg.2026.1707196

**Published:** 2026-02-24

**Authors:** Xi Xu, Xin Cao, Qian Wu

**Affiliations:** 1 College of Fine Arts, Fujian Normal University, Fuzhou, China; 2 School of Art and Design, Fuzhou University of International Studies and Trade, Fuzhou, China

**Keywords:** cognitive abilities, educational agents, learning outcomes, meta-analysis, non-cognitive abilities

## Abstract

**Introduction:**

With the deep integration of artificial intelligence technology in the field of education, educational agents as an intelligent teaching tool possessing interactive and personalised characteristics have drawn increasing attention for their impact on learning outcomes.

**Methods:**

This study employs a meta-analysis methodology to systematically synthesise 52 empirical investigations published in internationally authoritative journals between 2015 and 2025. It examines the overall effect of educational agents on student learning outcomes, their specific manifestations at cognitive and non-cognitive levels, and the influence of moderating variables such as types of agents, subjects, sample size, and academic level.

**Results:**

Findings indicate that educational agents exert a significant positive influence on student learning outcomes. Regarding cognitive abilities, they demonstrate moderate to substantial enhancement effects on creative thinking, academic performance, and communication skills, while their impact on spatial ability and problem-solving skills falls below statistical significance. Regarding non-cognitive abilities, learning motivation and learning attitude showed significant enhancement, whereas the effects on learning engagement and learning interest were smaller and non-significant. Moderation analyses indicated that the impact of educational agents was particularly pronounced among chatbots, universities, small-scale settings, and engineering technology disciplines.

**Discussion:**

This study reveals limitations in educational agents’ cultivation of complex abilities and personalised adaptation, providing empirical evidence for their precise application and optimised design.

## 1 Introduction

With the rapid advancement of artificial intelligence technology in the educational sphere, educational agents as intelligent teaching tools possessing interactive and personalised characteristics have progressively become pivotal media for driving digital transformation in education (Alam and Mohanty, 2023). These agents can simulate human teaching behaviours—such as providing real-time answers to queries, designing personalised learning pathways, and offering emotional support—to create adaptive learning environments for students. Their potential value in enhancing learning engagement and optimising knowledge acquisition efficiency has attracted significant academic attention (Sajja et al., 2024). In recent years, empirical research on educational agents has proliferated globally, spanning primary, secondary, tertiary, and vocational education across disciplines including mathematics, language learning, and science. This abundance of empirical evidence provides a rich foundation for examining their impact on student learning outcomes (Song and Wang, 2020). To demonstrate the impact of educational agents on learning outcomes, researchers have conducted numerous experimental or quasi-experimental studies, though conclusions vary considerably (Ma and Zhong, 2025). This study categorises these findings into the following three types:

Firstly, educational agents demonstrate a positive impact on students’ learning outcomes. Existing research generally indicates that such agents can enhance learning outcomes, self-efficacy, and digital literacy performance through immediate feedback and path planning (Chun et al., 2025; Wang et al., 2023), significantly improve academic performance (Alrakhawi et al., 2023), and effectively enhance non-cognitive abilities such as learning motivation (Tu et al., 2025). For instance, Sisman et al. (2021) examined the effects of educational robots on developing spatial abilities in 12-year-old students through a control-group comparison approach. Results indicated greater positive changes in spatial abilities among robotics course participants compared to non-participants. Boudouaia et al. (2024) compared the outcomes of undergraduate students using ChatGPT-4 versus receiving teacher-guided instruction for English writing learning, finding superior writing performance in the ChatGPT-4 group. Ait Baha et al. (2024) integrated AI-supported educational agents into programming education and conducted a comparative teaching experiment with 109 high school students. Findings indicated that the experimental group using educational agents demonstrated superior outcomes and enhanced teamwork capabilities.
Secondly, educational agents have no significant impact on student learning outcomes. For instance, Escalante et al. (2023) found no significant difference between ChatGPT-4-generated writing feedback and teacher feedback in influencing university students’ writing performance. Ortega-Ochoa et al. (2024) employed a quasi-experimental study and found that empathetic educational agents showed no significant difference from human teachers in promoting student learning, stimulating learning interest, or enhancing self-regulation abilities. Kahyaoğlu Erdoğmuş and Kurt (2024) investigated the cross-modal cognitive transfer characteristics of educational agents and found no significant differences across experimental groups in cognitive load, flow experience, or skill levels. Hwang and Zhang (2024) indicated that variations in role design and appearance of educational agents exerted no significant influence on learning motivation among secondary school pupils.
Thirdly, educational agents may exert negative influences on student learning outcomes. Some studies indicate that excessive intervention by such agents could suppress higher-order thinking, diminish social skills, and even impede the development of deep learning. Whether they can adequately fulfil the role of a teacher remains contentious (Dever et al., 2024; Lee et al., 2024). For instance, research by Bastani et al. (2024) demonstrated that while GAI-enabled educational agents improved high school students’ academic performance in the short term, they may also foster dependency and a lack of deep understanding, thereby hindering students’ long-term learning capacity development. A mixed-methods study by Lin and Yu (2025) on AI-enabled educational agents’ impact on language learning revealed that when agents’ linguistic styles proved indistinguishable from human teachers, students experienced anxiety and uncertainty regarding information quality, diminishing sustained learning motivation. Furthermore, Zhang et al. (2024) further observed that beyond the design of the educational agent itself, factors such as the learning environment and student gender may constrain its effectiveness in specific contexts, manifesting as cultural inappropriateness and diminished learning motivation.

Existing meta-analyses in AI and educational technology have laid important groundwork - confirming that AI tools can enhance learning outcomes (Wu and Yu, 2024; Hwang, 2022) and exploring the potential of generative AI (Ma and Zhong, 2025). However, three critical gaps remain. First, prior works focus on narrow subsets of tools (e.g., AI chatbots alone or broad AI tools) without comparing distinct educational agent subtypes, leaving unclear which agent designs are most effective. Second, they rely on aggregate outcome measures (e.g., academic performance or learning gains) that mask differential effects on specific cognitive and non-cognitive subdimensions, such as creative thinking versus spatial ability. Third, most prior meta-analyses report descriptive findings without linking results to theoretical frameworks, failing to explain why effects vary or provide actionable guidance for context-specific implementation.

In summary, existing research has examined the impact of educational agents on student learning outcomes from various perspectives. However, due to differences in independent variables, dependent variables, and moderating variables, research conclusions remain significantly divergent. This study anchors its hypotheses and analyses in four core theories of learning and AI in education, which guide the interpretation of how educational agents influence learning outcomes. Social Learning Theory (Bandura and Walters, 1977): Posits that learning occurs through observation, imitation, and social interaction. Cognitive Load Theory (Sweller and Chandler, 1991): Argues that effective learning depends on managing cognitive load, extraneous load (irrelevant stimuli) should be minimized, while intrinsic load (task complexity) and germane load (deep processing) are optimized. Technology Acceptance Model (Davis, 1989): Proposes that technology use is driven by perceived usefulness (belief that the tool improves performance) and perceived ease of use (low effort to operate). Self-Determination Theory (Ryan and Deci, 2024): Highlights three psychological needs, autonomy, competence, and relatedness, as drivers of intrinsic motivation.

Social Learning Theory primarily explains outcomes shaped by agentic role enactment and bidirectional interactivity (Bandura and Walters, 1977). The social presence of agents fosters observational learning and identification, activating psychological mechanisms of trust and social comparison. These mechanisms enhance non-cognitive outcomes such as learning motivation and attitude, as agents model achievable goals and provide interactive feedback. Cognitive Load Theory underpins outcomes influenced by the personalisation feature of agents (Sweller and Chandler, 1991). By tailoring content to individual progress and scaffolding complex tasks, agents reduce extraneous cognitive load and promote germane load, mechanisms that support cognitive outcomes including creative thinking, academic performance, and communication skills. The Technology Acceptance Model clarifies moderating effects related to perceived usefulness and ease of use (Davis, 1989). Agent features such as intuitive interaction and task relevance enhance these perceptions, particularly among university students and in small-class settings. This psychological mechanism of technology acceptance strengthens the overall impact of agents on learning outcomes across contexts. Self-Determination Theory explains outcomes linked to all three agent features (Ryan and Deci, 2024). Bidirectional interactivity supports relatedness, personalisation fosters competence, and agentic role enactment provides opportunities for autonomy. These mechanisms collectively drive intrinsic motivation, supporting learning engagement and interest—though weak activation of these mechanisms in some studies explains the marginally non-significant results for these outcomes.

**Figure 1:**
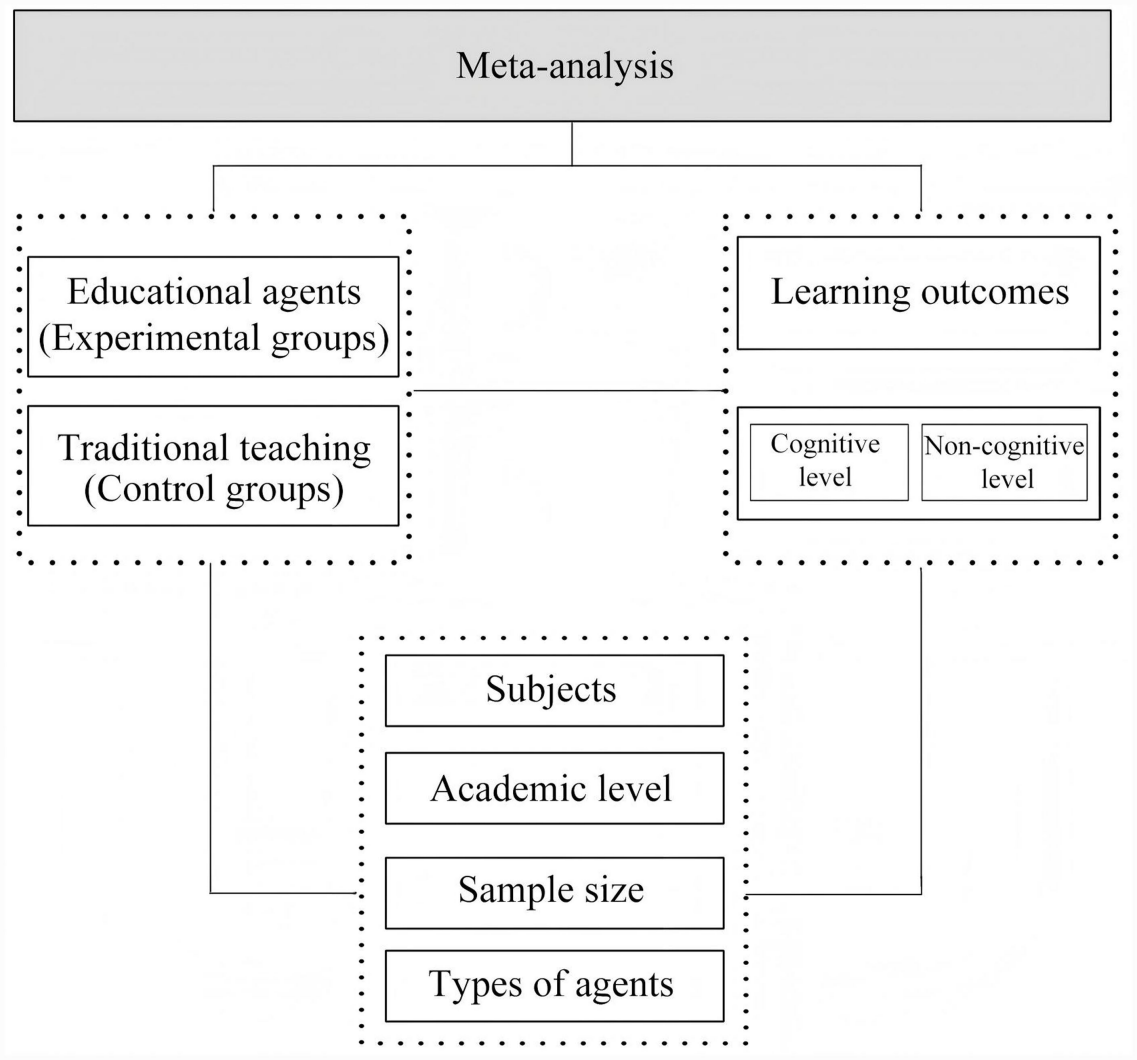
Research framework for meta-analysis.

In light of this, this study employs a meta-analysis methodology to screen and analyse empirical research literature from internationally authoritative databases between 2015 and 2025 concerning the influence of educational agents on student learning outcomes. As illustrated in Figure 1, this study seeks to address the following questions through meta-analysis:

RQ1: Can educational agents significantly enhance student learning outcomes compared to traditional learning methods?

RQ2: What is the extent of educational agents' influence on student learning outcomes across cognitive and non-cognitive dimensions?

RQ3: Do different types of educational agents yield varying impacts on student learning outcomes?

RQ4: Do different subjects influence the impact of educational agents on student learning outcomes?

RQ5: Do different sample sizes influence the impact of educational agents on student learning outcomes?

RQ6: Do different academic levels influence the impact of educational agents on student learning outcomes?

## 2 Materials and methods

This study strictly adhered to the PRISMA guidelines for literature retrieval and selection (Moher et al., 2009), and followed the fundamental meta-analysis workflow proposed by Borenstein et al. (2021): literature search, literature screening, literature coding, calculation of the average effect size, calculation of the effect size for moderating variables, and comprehensive discussion of the research findings.

### 2.1 Operational definition of educational agents

To ensure consistency in literature screening, this study defined educational agents based on three mutually inclusive core criteria, drawing on Yusuf et al. (2025) and Zhang et al. (2024)—leading frameworks for agent classification in educational technology research: Bidirectional interactivity refers to the agent can initiate or respond to student inputs (verbal, text, or behavioral) in real time, rather than providing one-way content delivery. For example, a chatbot that answers follow-up questions qualifies, while a pre-recorded AI lecture platform does not. Personalised adaptation refers to the agent uses student data (e.g., test scores, learning behavior, preferences) to adjust its functions, such as tailoring practice tasks for low-performing students or adapting feedback tone for anxious learners. Static tools with fixed content (e.g., generic AI flashcards) are excluded. Agentic role enactment refers to the agent operates as a distinct social or functional actor (e.g., a virtual tutor, peer collaborator, or motivational mentor) with a defined role in the learning process. Tools that lack role identity (e.g., AI grammar checkers without interactive guidance) are excluded.

This definition explicitly differentiates educational agents from other AI tools: For instance, a non-interactive AI grading system is excluded (fails criterion 1), a one-size-fits-all educational game is excluded (fails criterion 2), and a static AI textbook is excluded (fails all three criteria).

### 2.2 Handling of borderline cases

During literature screening, 17 studies were identified as borderline cases (i.e., meeting 1–2 core criteria but not all). To ensure consistent classification, we implemented a two-stage evaluation process: Two researchers (with expertise in educational agents) evaluated each borderline case against the three core criteria. For each study, they documented: (a) which criteria were met/unmet; (b) key evidence from the manuscript (e.g., tool adapts to student progress but lacks bidirectional interaction); (c) a preliminary inclusion/exclusion recommendation: Initial agreement between researchers was 82% (14/17 cases). The three discrepant cases were resolved via: (a) re-examining the study’s description of tool functions; (b) cross-referencing with the operational definition; (c) consultation with a third expert (a professor specializing in educational AI with 10 + years of agent research).

Included (*n* = 5): Studies met all three criteria upon re-evaluation (e.g., a gamified agent that interacts via text, adapts tasks to performance, and acts as a “peer learner”). Excluded (*n* = 12): Studies failed to meet at least one core criterion (e.g., a tool that personalizes content but only provides one-way feedback, failing criterion 1, a chatbot that interacts but has no adaptive functions, failing criterion 2).

### 2.3 Selecting the variables

This study primarily investigates the potential impact and fundamental characteristics of educational agents on student learning outcomes. Employing the classification framework for educational agents proposed by Yusuf et al. (2025), and drawing upon the literature reviewed in this research, educational agents are categorised into intelligent learning systems, chatbots, and gamified learning agents.

Different studies focus on varying types of learning outcomes. Some research centres on cognitive dimensions (such as creative thinking, learning gains, spatial abilities, etc.), while other studies emphasise non-cognitive dimensions (such as learning perseverance, learning motivation, etc.). These evaluation criteria themselves exhibit fundamental differences in their evaluation methods and pathways to realisation. Therefore, this study references Vittadini et al. (2022) on the structure of human capabilities, defining learning performance as students’ comprehensive outcomes under the interventions of educational agent, encompassing both cognitive and non-cognitive dimensions. Cognitive abilities are subdivided into five categories based on Anderson’s (2013) framework: firstly, creative thinking, denoting the generation of novel and useful ideas; secondly, academic performance, referring to knowledge or skills acquired through learning; thirdly, spatial ability, involving the capacity to perceive and process spatial relationships; fourthly, problem-solving skills, denoting the ability to integrate knowledge and make judgements in complex situations; and fifthly, communication skills, referring to the capacity for knowledge exchange, viewpoint expression, and collaborative interaction. Non-cognitive abilities, following Kautz et al. (2014), encompass learning motivation, learning attitude, learning engagement, and learning interest, emphasising their regulatory role in learning behaviours and deep processing capabilities.

Moreover, variations in learning outcomes following the use of educational agents differ across students of varying academic levels and sample sizes. In engineering and science-oriented tasks, educational agents demonstrate greater efficacy in providing cognitive support or pathway recommendations (Wang et al., 2025); conversely, within comprehensive curricula, their effectiveness frequently hinges upon individual differences and interaction modalities (George, 2023). Consequently, this study introduces academic level, sample size, and subjects as moderating variables.

### 2.4 Literature search

This study employed the keywords “educational agents” and “learning outcomes”, conducting literature searches in the scientific databases Web of Science, Google Scholar, and Scopus in strict accordance with the PRISMA guidelines. PRISMA provides detailed operational standards applicable to most other types of systematic review research (Page et al., 2021). Keywords included “educational agents”, “chatbot”, “generative AI agent”, “artificial intelligence”, “teaching agents”, “learning”, “study outcomes”, and “learning effect.” Journal articles published between 2015 and July 2025 were retrieved from the aforementioned scientific databases. All selected articles originated from internationally authoritative journals indexed in SSCI, SCI, or AHCI. The search yielded 2,784 qualifying publications: 1,349 from Google Scholar, 953 from Web of Science, and 482 from Scopus.

### 2.5 Literature screening

As the retrieved literature did not fully meet the requirements, it was necessary to screen the studies. This research established the following selection criteria: (1) The research content must address the impact of educational agents on learning outcomes; (2) The research methodology must be experimental or quasi-experimental, with the independent variable being the use of educational agents (defined as tools meeting three core criteria: bidirectional interactivity, personalised adaptation, agentic role enactment, see Section 2.1) during the learning process, and the dependent variable being learning outcomes; (3) The study included both experimental and control groups to compare differences in learning performance; (4) The research findings included the sample size, mean value, and standard deviation.

**Figure 2:**
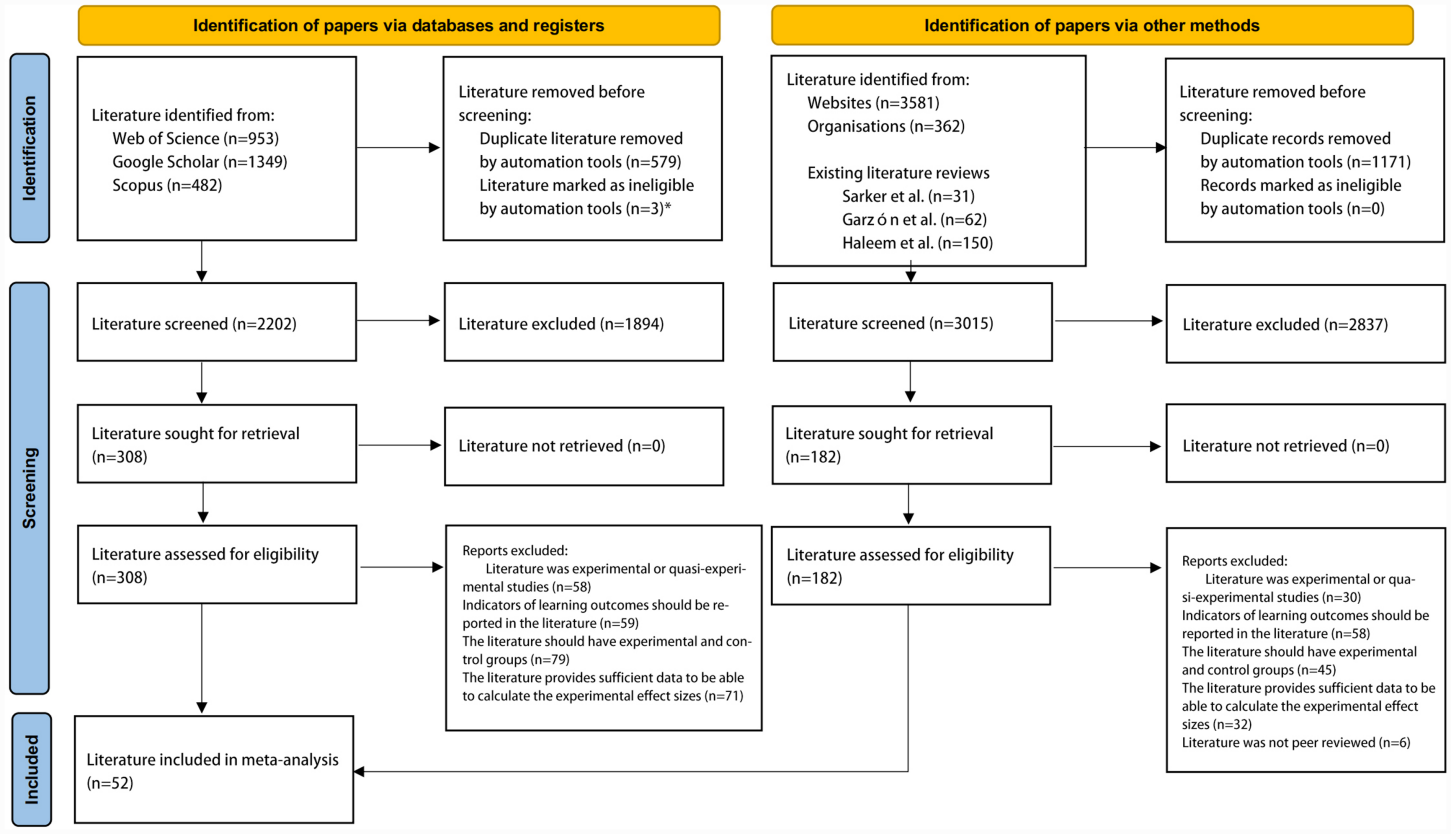
PRISMA process for literature screening.

The 2,784 retrieved literature records were imported into EndNote. After removing duplicates, 837 literature records remained following the initial screening. Two experts in the field of educational agent then conducted a double screening of the literature based on subject and abstract. The reliability of each screening process was assessed using Cohen’s Kappa consistency test, yielding Kappa coefficients of 0.88, 0.91, 0.92, and 0.89 respectively—all exceeding 0.8 (indicating near-complete agreement). This demonstrates the screening results possess good reliability (Borenstein et al., 2021). Following this screening, 48 literature items remained. The snowball sampling method was employed to trace both forward and backward citations from these selected literature, thereby identifying additional relevant literature (Lim et al., 2018). This approach yielded four additional literature, resulting in a final dataset of 52 literature, as illustrated in Figure 2.

### 2.6 Literature coding

The learning outcomes of educational agents may be influenced not only by the types of agents employed but also by moderating variables such as sample size, academic level, and subjects (Garzón and Acevedo, 2019; Zhang et al., 2020). Therefore, alongside coding for learning outcomes and group assignments, these moderating variables also require coding. For sample size coding, the classification into small scale (b1 = 1–50), medium scale (b2 = 51–100), and large scale (b3 = over 100) is based on established conventions in educational technology meta-analyses, specifically following the framework proposed by Hwang (2022). This classification is widely adopted in similar syntheses due to its alignment with typical class size distributions in international educational contexts and its ability to capture meaningful variations in the impact of technology interventions. Moreover, variations in learning outcomes following the use of educational agents differ across students of varying academic levels and sample sizes. The sample size classification employed in this study is consistent with prior meta-analyses examining technology in education, ensuring comparability with existing literature and enhancing the relevance of the moderating analysis. In engineering and science-oriented tasks, educational agents demonstrate greater efficacy in providing cognitive support or pathway recommendations; conversely, within comprehensive curricula, their effectiveness frequently hinges upon individual differences and interaction modalities. Consequently, this study introduces academic level, sample size, and subjects as moderating variables.

**Figure 3:**
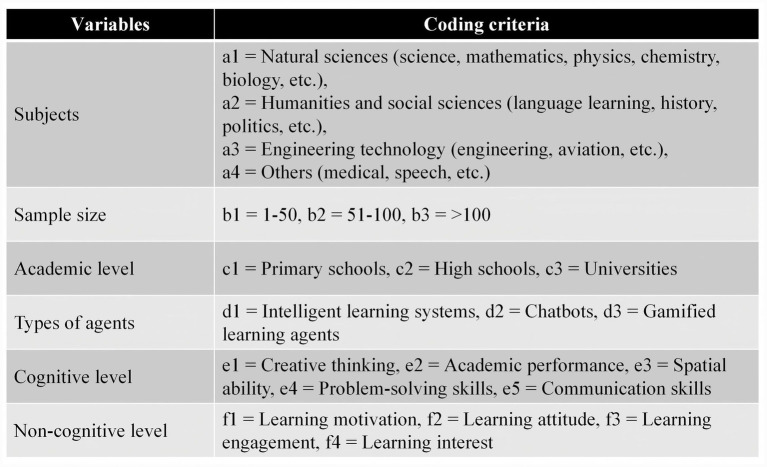
Literature coding criteria.

Two researchers specialising in educational agents negotiated consensus on coding dimensions and categories. Fifty-two studies underwent independent back-to-back coding, with acceptable coding consistency verified (Cohen’s Kappa = 0.907). The specific coding methodology is illustrated in Figure 3.

### 2.7 Data analysis

This study employed Comprehensive Meta-Analysis (CMA) Version 3 to input the sample size, mean, and standard deviation from the original research. Using these three values, the effect size for each study was calculated, and the magnitude of influence was assessed through the effect size. The meta-analysis primarily utilised the fixed-effects model and random-effects model proposed by Borenstein et al. (2021). Literature review revealed that the relationship between educational agents and learning outcomes may be influenced by complex factors including types of agents, sample sizes, academic levels, and subjects. Given the variability in study designs, this research employed a random-effects model to estimate the overall effect size. The impact of educational agents on student learning outcomes was explored through tests for publication bias and heterogeneity, analysis of the overall effect value, and examination of moderating variables (Qiu et al., 2024).

## 3 Results

### 3.1 Publication bias test

**Figure 4:**
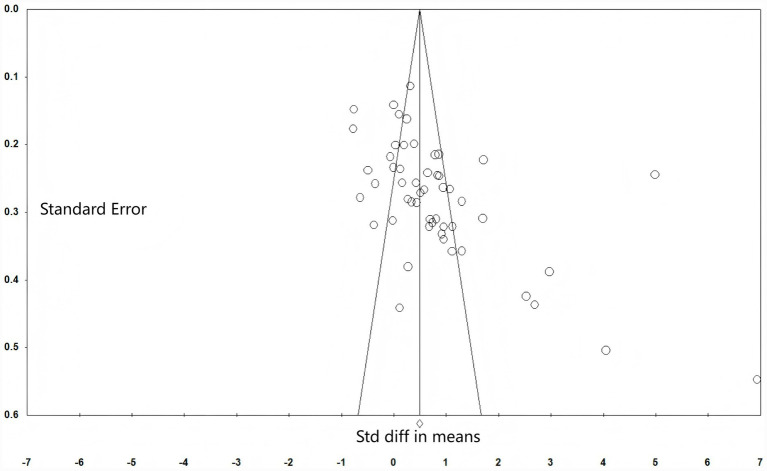
Funnel scatter graphic.

In meta-analyses, publication bias may arise when the selected samples fail to represent the research population within the field, thereby compromising the accuracy of findings (Thornton and Lee, 2000). To assess whether publication bias exists in this study, a comprehensive evaluation method combining funnel plots, Begg’s test, and the classic fail-safe *N* was employed. As shown in Figure 4, the horizontal axis of the funnel plot represents the mean effect size, while the vertical axis denotes the standard deviation of effect sizes. Visualising publication bias through the funnel plot reveals that the effect sizes of 52 independent studies predominantly cluster in the upper half of the plot, distributed symmetrically around the mean effect size. This indicates a minimal likelihood of publication bias. Begg’s test results indicate: *T* = 0.39 < 1.96, *p* < 0.001, further substantiating the absence of bias.

This study also employed the Classic fail-safe N method proposed by Rosenthal (1979) to assess publication bias. The Classic fail-safe N serves as another effective indicator for evaluating the occurrence of publication bias. This coefficient emphasises that when meta-analysis results are statistically significant, a larger coefficient value indicates a lower probability of conclusion reversal, thereby reducing the likelihood of publication bias. Specifically, publication bias is deemed present if the fail-safe index falls below 5*n* + 10, where n represents the number of studies included in the meta-analysis. As shown in Table 1, this study’s fail-safe index of 4,652 substantially exceeds 270 (5 × 52 + 10), indicating a minimal likelihood of publication bias. Based on a comprehensive assessment of the two evaluation methods, the fundamental findings of this study are considered reliable and capable of yielding stable conclusions.

**Table 1:** Results of classic fail-safe *N*.

Variable	Value
*Z*-value for observed studies	18.64
*p*-value for observed studies	0.00
Alpha	0.05
Tails	2.00
*Z* for alpha	1.95
Number of observed studies	52
Number of missing studies that would bring *p*-value to > alpha	4,652

### 3.2 Heterogeneity test

Heterogeneity tests are primarily used to measure the degree of variation in effect sizes across studies, aiming to determine whether the results of independent studies are combinable (Petitti, 2001). If heterogeneity exists between studies, a random-effects model is employed; if no heterogeneity is present, a fixed-effects model is used (Rücker et al., 2008). As shown in Table 2, this study primarily employed *Q* and *I*^2^ for testing. Results indicate *Q* = 930.50, *p* < 0.001, confirming heterogeneity among study samples. The *I*^2^ value of 94.51% exceeds 75%, indicating that 94% of heterogeneity stems from genuine differences in effect sizes, with only 6% attributable to systematic error. Factors such as publication year, country of origin, sample size, and study subjects across the included studies may contribute to this heterogeneity. The I^2^ value reflects the proportion of variance in effect sizes attributable to heterogeneity, a higher value indicates stronger heterogeneity. This study employed a random-effects model for analysis to ensure the validity and reliability of the results, thereby effectively accounting for heterogeneity.

**Table 2:** Heterogeneity test results.

Effect model	Combined effect size	95% CI		Two-tailed test	Heterogeneity test			
		Upper limit	Lower limit	*p*	*Q*	*I^2^*	*df*	*p*
Fixed effects model (FEM)	0.49	0.42	0.55	<0.0001	930.50	94.51%	51	<0.0001
Random effects model (REM)	0.82	0.54	1.11					

### 3.3 Sensitivity test

Sensitivity analysis is primarily employed to examine outliers that may influence the overall effect size (Copas and Shi, 2000). This study utilised a One-Study Removal Analysis to assess the impact of extreme positive and negative effect sizes on the overall effect size. In this study, the effect size within the 95% confidence interval remained 0.42–0.55 (fixed-effects model) and 0.54–1.11 (random-effects model) after removing any single study. Consequently, the removal of any single study did not affect the overall effect size. This also fully demonstrates that the meta-analysis results obtained in this study are highly robust.

### 3.4 Effect size analysis

**Figure 5:**
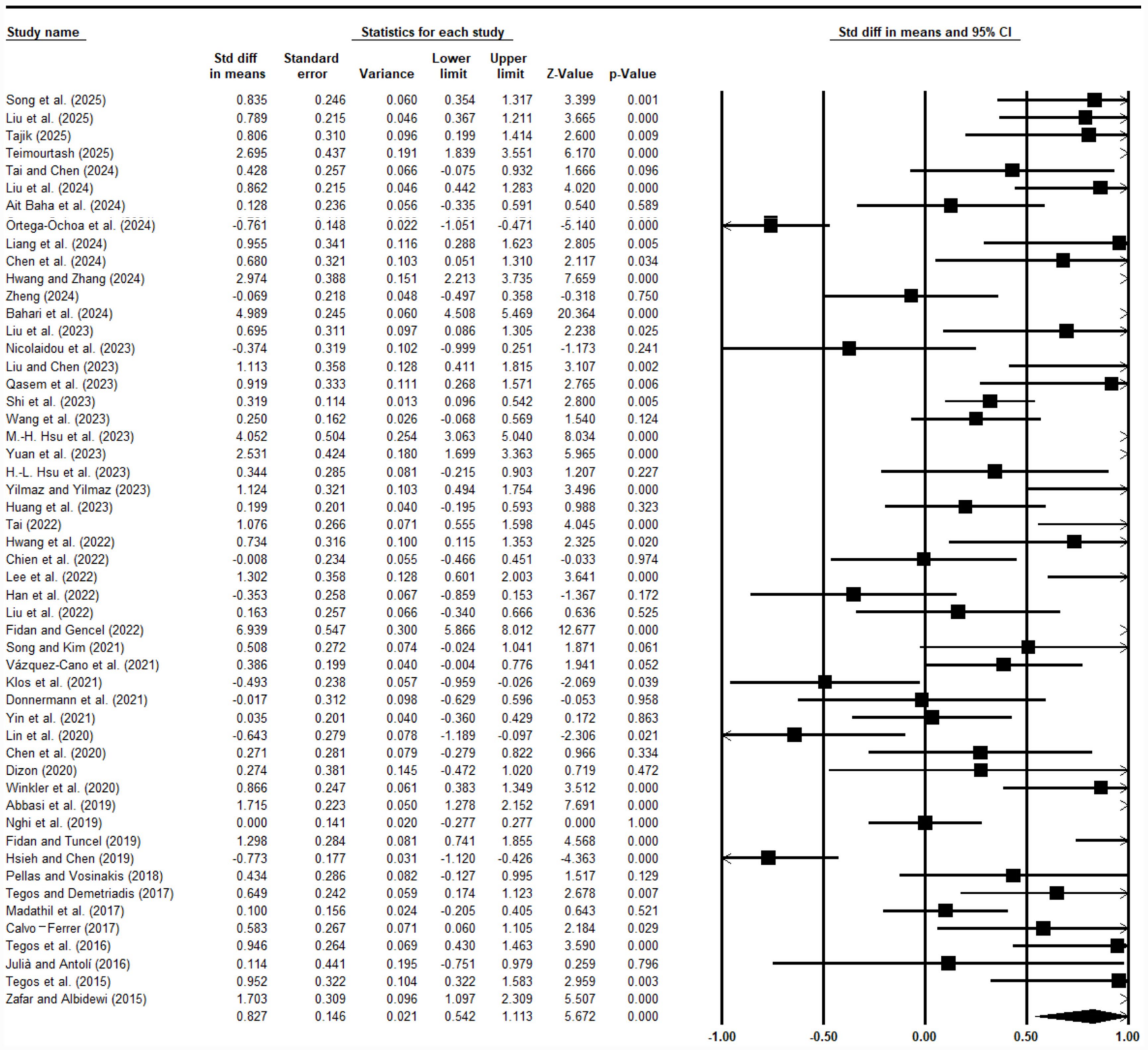
Forest map of the original research.

This study employed a random-effects model to estimate the overall impact of educational agents on student learning performance. Data including sample sizes, means, and standard deviations from pre- and post-tests of experimental and control groups across 52 studies were input into the meta-analysis software CMA 3.0. The overall effect size for educational agents’ influence on learning outcomes is presented in Figure 5. Following Borenstein et al. (2021), an effect size below 0.2 indicates a small effect, 0.2 or above but below 0.5 denotes a small-to-moderate effect, 0.5 represents a moderate effect, 0.5 or above but below 0.8 signifies a large-to-moderate effect, and 0.8 or above indicates a large effect. The overall effect size was 0.827, with *p* < 0.001, indicating that educational agents exert a large positive effect on student learning outcomes. Results are deemed statistically significant at the *α* = 0.05 threshold. *p*-values between 0.05 and 0.10 are described as marginally non-significant to reflect weak directional trends, but these do not meet conventional standards for meaningful effects. Exact *p*-values, 95% confidence intervals, and test statistics are provided for all key results to enable transparent evaluation of robustness.

The impact of educational agents on different categories of learning performance is shown in Table 3. Educational agents exerted a significant positive effect on both students’ cognitive abilities (effect size = 0.81, *p* < 0.001) and non-cognitive abilities (effect size = 0.79, *p* < 0.001). The results of the between-group effect test were non-significant (*p* > 0.05), indicating that the educational agents had no significant difference in its impact on the two types of learning outcomes.

**Table 3:** Analysis of the impact of educational agents on cognitive and non-cognitive abilities.

Types of learning outcomes	*N*	Effect size	95% CI		Heterogeneity test *I*^2^ (%)	*Z*	*p*	Between-group effect size
			Upper limit	Lower limit				
Cognitive abilities	38	0.81	0.52	1.10	83.53	3.74	<0.001	*Chi*^2^ = 0.73, *p* = 0.12
Non-cognitive abilities	32	0.79	0.48	1.12	91.46	3.78	<0.001	
Total	52	0.82	0.53	1.11	94.51	5.61	<0.001	

Subsequently, this study further examined the impact of educational agents on student learning outcomes by integrating specific criteria of cognitive and non-cognitive abilities from the analysed literature, as presented in Table 4. At the cognitive level, educational agents demonstrated a substantial positive effect on students’ creative thinking (*d* = 0.89, 95% *CI* = 0.51–1.27, *Z* = 4.56, *p* < 0.001), a moderately large positive effect on academic performance (*d* = 0.72, 95% *CI* = 0.43–1.01, *Z* = 4.82, *p* < 0.001), and a moderately large positive effect on communication skills (*d* = 0.75, 95% *CI* = 0.38–1.12, *Z* = 3.98, *p* < 0.001). At the cognitive level, the substantial positive effect of agents on creative thinking is best explained by Cognitive Load Theory. The personalisation feature of agents reduces extraneous load by targeting individual learning gaps, while bidirectional interactivity promotes germane load through adaptive feedback. Together, these mechanisms free cognitive resources for divergent thinking, consistent with the observed effect size. Academic performance and communication skills similarly reflect Cognitive Load Theory and Social Learning Theory, respectively. Personalisation minimises irrelevant information to support knowledge acquisition, while the social presence of agents (from role enactment) creates a safe environment for practicing communication—activating trust and observational learning mechanisms. Concurrently, educational agents showed a weak, marginally non-significant positive trend for spatial ability (*d* = 0.38, 95% *CI* = −0.02–0.78, *p* = 0.06) and problem-solving skills (*d* = 0.35, 95% *CI* = −0.03–0.73, *p* = 0.08). These *p*-values are near the conventional *α* = 0.05 threshold for significance, indicating a weak directional trend, but they do not meet standards for definitive statistical significance, cannot conclude educational agents meaningfully improve these outcomes. The between-group effect size test yielded significant results (*p* < 0.05), indicating statistically meaningful differences in the educational agent’s impact across cognitive ability dimensions. It should be noted that the higher effect size observed for students’ creative thinking may be influenced by factors such as the limited number of studies included and the heterogeneity of measurement tools. The marginally non-significant trends for spatial ability and problem-solving skills stem from incomplete activation of theoretical mechanisms. For spatial ability, most agents lacked dynamic interaction features needed to reduce intrinsic load (Cognitive Load Theory), limiting the development of spatial processing. For problem-solving skills, agents often failed to scaffold autonomy (Self-Determination Theory) or model problem-breaking strategies (Social Learning Theory), meaning key psychological mechanisms were not sufficiently triggered. At the non-cognitive level, educational agents exerted a moderately small positive effect on both learning motivation (*d* = 0.45, 95% *CI* = 0.21–0.69, *Z* = 3.68, *p* < 0.001) and learning attitude (*d* = 0.42, 95% *CI* = 0.18–0.66, *Z* = 3.42, *p* < 0.001). Concurrently, educational agents showed a weak, marginally non-significant positive trend for learning engagement (*d* = 0.28, 95% *CI* = −0.01–0.57, *p* = 0.07) and learning interest (*d* = 0.25, 95% *CI* = −0.02–0.52, *p* = 0.09). These near-significant p-values reflect a weak directional trend but do not meet the *α* = 0.05 threshold for statistical significance—unlike the fully significant effects observed for learning motivation (*p* < 0.001) and attitude (*p* < 0.001), which are robust. The between-group effect test yielded non-significant results (*p* > 0.05), indicating no significant differences in the effects of the educational agents across the various dimensions of non-cognitive abilities. At the non-cognitive level, significant effects for learning motivation and attitude align with Social Learning Theory and Self-Determination Theory. The social presence of agents (role enactment) fosters relatedness, while personalised feedback (personalisation) enhances competence—both mechanisms driving motivation and positive attitudes. The marginally non-significant results for learning engagement and interest reflect weak activation of autonomy support (Self-Determination Theory). Most agents provided fixed tasks rather than enabling student choice, meaning the psychological mechanism of autonomy was not adequately stimulated, limiting sustained engagement.

**Table 4:** Analysis of the impact of educational agents on various dimensions of cognitive and non-cognitive abilities.

Capability level	Specific criteria	*N*	Effect size	95% CI		Heterogeneity test *I*^2^ (%)	*Z*	*p*	Between-group effect size
				Upper limit	Lower limit				
Cognitive abilities	Creative thinking	15	0.89	0.51	1.27	92.62	4.56	<0.001	*Chi*^2^ = 12.85, *p* = 0.01
	Academic performance	28	0.72	0.43	1.01	91.73	4.82	<0.001	
	Communication skills	12	0.75	0.38	1.12	91.74	3.98	<0.001	
	Spatial ability	9	0.38	-0.02	0.78	89.69	1.88	0.06	
	Problem-solving skills	16	0.35	-0.03	0.73	90.43	1.75	0.08	
Non-cognitive abilities	Learning motivation	22	0.45	0.21	0.69	90.17	3.68	<0.001	*Chi*^2^ = 5.23, *p* = 0.15
	Learning attitude	19	0.42	0.18	0.66	89.40	3.42	<0.001	
	Learning engagement	14	0.28	−0.01	0.57	89.44	1.81	0.07	
	Learning interest	17	0.25	-0.02	0.52	89.34	1.70	0.09	

### 3.5 Moderator variables analysis

#### 3.5.1 Sample size

To assess the applicability of educational agents across different class sizes, this study categorised the sample according to class size into small-scale (1–50 pupils), medium-scale (50–100 pupils), and large-scale (over 100 pupils) groups. Detailed analysis is presented in Table 5.

**Table 5:** Differences in the impact of different sample size.

Sample size	*N*	Effect size	95% CI		Heterogeneity test *I*^2^ (%)	*Z*	*p*	Between-group effect size
			Upper limit	Lower limit				
1–50	10	0.90	0.43	1.37	86.07	3.74	<0.001	*Chi*^2^ = 0.49, *p* = 0.78
50–100	20	0.85	0.41	1.30	92.13	3.78	<0.001	
>100	22	0.62	-0.01	1.27	98.20	1.90	0.05	
Total	52	0.82	0.53	1.11	94.51	5.61	<0.001	

As shown in Table 5, the between-group heterogeneity test for sample size yielded *Chi*^2^ = 0.49, *df* = 2, *p* = 0.78 (not significant). While effect sizes trended downward from small (*d* = 0.90) to medium (*d* = 0.85) to large classes (*d* = 0.62), the non-significant between-group test confirms this variation does not meet the *α* = 0.05 threshold for statistical meaningfulness. This does not indicate stable influence across class sizes, only that group differences are not statistically robust. Data results for small-scale (effect size = 0.90, *Z* = 3.74, *p* < 0.001), medium-scale (effect size = 0.85, *Z* = 3.78, *p* < 0.001), and large-scale (effect size = 0.62, *Z* = 1.90, *p* = 0.05) classes. These findings demonstrate that the educational agents exerts a positive, facilitating influence on students across all class sizes. The impact is most pronounced for small-scale classes, followed by medium-scale classes, with the effect being relatively smallest for large-scale classes. Significant differences were observed between small-scale and medium-scale classes.

#### 3.5.2 Academic level

To assess the applicability of educational agents across different academic levels, this study categorised the sample into primary schools, high schools, and universities. The detailed analysis is presented in Table 6.

**Table 6:** Differences in the impact of different academic level.

Academic level	*N*	Effect size	95% CI		Heterogeneity test *I*^2^ (%)	*Z*	*p*	Between-group effect size
			Upper limit	Lower limit				
Primary Schools	8	0.64	−0.09	1.39	28.18	1.70	0.08	*Chi*^2^ = 1.45, *p* = 0.48
High Schools	9	0.51	−0.17	1.21	92.14	1.47	0.14	
Universities	35	0.95	0.59	1.30	95.75	5.26	<0.001	
Total	52	0.82	0.45	1.14	94.51	4.52	<0.001	

As shown in Table 6, the between-group heterogeneity test for academic level yielded *Chi*^2^ = 1.45, *df* = 2, *p* = 0.48 (not significant). University students showed a larger effect size (*d* = 0.95) compared to primary (*d* = 0.64) and high school students (*d* = 0.51), but the non-significant between-group test means this trend is not statistically reliable at the α = 0.05 threshold, cannot confirm stable influence across levels, only that group differences are not statistically meaningful. Primary schools (effect size = 0.64, *Z* = 1.70, *p* = 0.08), high schools (effect size = 0.51, *Z* = 1.47, *p* = 0.14), and universities (effect size = 0.95, *Z* = 5.26, *p* < 0.001) indicate that the educational agents positively promotes learning outcomes for students across all three levels. The impact is most pronounced for university students, followed by primary school pupils, with the effect being relatively smallest for secondary school students. Only the learning improvement observed among university students reached a statistically significant level.

#### 3.5.3 Subjects

To assess the applicability of the educational agents across different subjects, as illustrated in Figure 3, this study categorised the samples into four groups based on subjects. The detailed analysis results are presented in Table 7.

**Table 7:** Differences in the impact of different subjects.

Subjects	*N*	Effect size	95% CI		Heterogeneity test *I*^2^ (%)	*Z*	*p*	Between-group effect size
			Upper limit	Lower limit				
Natural sciences	27	0.76	0.36	1.17	89.13	3.71	<0.001	*Chi*^2^ = 1.73, *p* = 0.62
Humanities and social sciences	19	0.75	0.26	1.23	96.40	3.04	0.002	
Engineering technology	2	1.33	−0.22	2.88	93.58	1.67	0.09	
Others	4	1.41	0.34	2.48	98.18	2.59	0.01	
Total	52	0.82	0.49	1.21	94.51	4.68	<0.001	

As shown in Table 7, the between-group effect yielded *Chi*^2^ = 1.73, *p* = 0.62, indicating that the intergroup difference lacked statistical significance. Consequently, the educational agent’s impact on different subjects proved relatively stable, with no significant variation observed. Data results for natural sciences (effect size = 0.76, *Z* = 3.71, *p* < 0.001), humanities and social sciences (effect size = 0.75, *Z* = 3.04, *p* = 0.002), engineering technology (effect size = 1.33, *Z* = 1.67, *p* = 0.09), and others (effect size = 1.41, *Z* = 4.68, *p* = 0.01) indicate that the educational agents exerts a positive, facilitating influence on the learning outcomes of students across all four subject areas. Students in the others category experienced the greatest impact on learning effectiveness, followed by those in engineering technology, then natural sciences, with humanities and social sciences students showing the relatively smallest effect. The degree of learning effectiveness enhancement for natural sciences, humanities and social sciences, and others students all reached a level of significant difference.

#### 3.5.4 Types of agents

In accordance with the coding table specifications, this study conducted a comparative analysis of the impact of different types of agents on student learning outcomes. The specific results are presented in Table 8.

**Table 8:** Differences in the impact of different types of agents.

Types of agents	*N*	Effect size	95% CI		Heterogeneity test *I*^2^ (%)	*Z*	*p*	Between-group effect size
			Upper limit	Lower limit				
Intelligent learning systems	10	0.41	−0.24	1.07	88.71	1.24	0.21	*Chi*^2^ = 1.97, *p* = 0.37
Chatbots	37	0.94	0.60	1.29	95.31	5.36	<0.001	
Gamified learning agents	5	0.80	−0.13	1.73	93.14	1.67	0.09	
Total	52	0.82	0.36	1.18	94.51	3.68	<0.001	

As shown in Table 8, the effect sizes for the educational agents across the three types of agents were 0.41, 0.94, and 0.80 respectively, with a combined effect size of 0.82. A two-tailed test (*p* < 0.001) indicates that all three types of agents exert a positive, promoting effect on student learning outcomes. The between-group heterogeneity test for agent type yielded *Chi*^2^ = 1.97, *df* = 2, *p* = 0.37 (not significant). Chatbots trended toward a larger effect size (*d* = 0.94) compared to gamified learning agents (*d* = 0.80) and intelligent learning systems (*d* = 0.41), but the non-significant test result indicates these differences do not meet the *α* = 0.05 threshold for statistical significance. This does not mean the impact is stable across agent types, only that cannot definitively conclude agent type systematically alters effectiveness based on current data. Among these, chatbots exhibited the most pronounced effect, followed by gamified learning agents, with intelligent learning systems ranking last. Only the learning outcomes of students using chatbots demonstrated a statistically significant level of improvement.

## 4 Discussion

This study employed a meta-analysis methodology to systematically review and analyse 52 international publications from 2015 to 2025 concerning the impact of educational agents on student learning outcomes. The findings indicate:

1) Educational agents exert a significant positive influence on students’ learning outcomes (effect size = 0.82, p < 0.001). At both cognitive (effect size = 0.81) and non-cognitive (effect size = 0.79) levels, results revealed no significant differences between the two domains (*p* > 0.05). This indicates that educational agents enhances learning outcomes across both cognitive and non-cognitive dimensions. This finding aligns closely with the conclusions of Wang et al. (2023), who demonstrated that intelligent teaching tools can simultaneously enhance both knowledge acquisition and learning motivation. This study further refined the specific criteria effects of cognitive and non-cognitive dimensions, addressing the limitation of previous research that focused solely on overall effects. At the cognitive level, the most pronounced promotional effects were observed for creative thinking (effect size = 0.89), academic performance (effect size = 0.72), and communication skills (effect size = 0.75). Conversely, spatial ability (effect size = 0.38) and problem-solving skills (effect size = 0.35) demonstrated modestly positive effects that failed to reach statistical significance. Spatial ability studies (*n* = 9) had small average sample sizes (*M* = 32 participants per study), leading to low statistical power (Cohen, 1992). For example, Sisman et al. (2021) included only 24 students in their robotics intervention, limiting the ability to detect small-to-moderate effects. Problem-solving studies (*n* = 16) predominantly focused on short-term interventions (≤4 weeks), while complex problem-solving requires sustained practice (García-Penalvo, 2023). Agents were often used for isolated tasks (e.g., single-step math problems) rather than multi-step, interdisciplinary challenges, failing to activate deep problem-solving processes. This aligns with Boudouaia et al.’s (2024) findings that ChatGPT-4 enhances English writing performance. Building upon this, the present study highlights the potential confounding influence of sample size on these outcomes. From the non-cognitive level, the promotional effects of both learning motivation (effect size = 0.45) and learning attitude (effect size = 0.42) were significant. Conversely, learning engagement (effect size = 0.28) and learning interest (effect size = 0.25) exhibited small positive effects that did not reach statistical significance. This partially aligns with findings by Lin and Yu (2025), who suggested that stylistic differences between AI agents and human teachers may induce student anxiety and diminish sustained interest. The present study further supplements this by highlighting the challenge of standardised AI agent designs failing to accommodate individualised preferences, thereby enriching the explanatory framework for non-cognitive influencing factors. The operational definition of educational agents (interactive, personalised, agentic) ensured that the included studies focused on tools with genuine collaborative potential—consistent with the large positive effect size (*d* = 0.82) observed. Excluding non-agent AI tools (e.g., static platforms) avoided diluting the analysis with tools that lack the core functions driving learning gains (Yusuf et al., 2025). The large overall effect of agents on learning outcomes reflects the combined activation of mechanisms from all four theories. Cognitive outcomes benefit most from Cognitive Load Theory-driven processes (reduced extraneous load), while non-cognitive outcomes are primarily shaped by Social Learning Theory (social presence) and Self-Determination Theory (need satisfaction). This alignment explains why creative thinking (strong Cognitive Load Theory activation) and learning motivation (strong Social Learning Theory and Self-Determination Theory activation) show the most robust effects.
2) To investigate the impact of types of educational agents on student learning outcomes, this study analysed three distinct categories: intelligent learning systems (effect size = 0.41), chatbots (effect size = 0.94), and gamified learning agents (effect size = 0.80). Results indicate no significant differences between these types (*p* > 0.05), with all enhancing student learning effectiveness. This aligns with Yusuf et al. (2025) that diverse pedagogical AI agents possess educational value, while providing more precise comparative effect sizes. Furthermore, this study found that educational agents did not significantly enhance students’ problem-solving abilities. In 62.5% of problem-solving studies, agents were used as answer providers rather than process guides, providing direct solutions instead of scaffolding (e.g., prompting students to break down problems). This reduces the agents’ ability to foster problem-solving strategies. Spatial ability interventions (e.g., educational robots) were rarely aligned with classroom curricula (e.g., disconnected from geometry lessons), leading to fragmented learning experiences that did not reinforce spatial skills (Zhang et al., 2024). This contrasts with the conclusion by Kahyaoğlu Erdoğmuş and Kurt (2024) that gamified agents can increase cognitive load and flow experiences. This discrepancy may arise because the problem-solving tasks included in this study were predominantly complex, interdisciplinary challenges, for which the agents provided insufficient depth of support. Agents reduce extraneous load by personalizing content (e.g., adapting to learning gaps) and scaffolding tasks, freeing cognitive resources for deep processing—explaining the significant effects on creative thinking (*d* = 0.89) and academic performance (*d* = 0.72). For example, chatbots that provide targeted feedback (rather than generic explanations) minimize irrelevant information, enabling students to focus on creative problem-solving (Boudouaia et al., 2024). Agents’ social presence (e.g., gamified mentors that “celebrate progress”) fosters observational learning and identification, enhancing non-cognitive outcomes like learning motivation (*d* = 0.45) and attitude (*d* = 0.42). This aligns with Bandura and Walters’s (1977) assertion that social models boost motivation by demonstrating achievable goals (Zhang et al., 2024).
3) In terms of subjects, the educational agents exerted a positive influence on the learning outcomes of students across all subjects, though the degree of impact showed no significant differences (*p* > 0.05). Notably, the educational agents demonstrated the most pronounced learning effect among students in the “others” course. This finding diverges from Saraç’s (2018) research outcomes. This discrepancy may stem from the application of the educational agents within the medical course, where it concretises abstract human organ modelling problems, thereby enhancing students’ comprehension of the subject matter. Concurrently, this approach may heighten students’ engagement and interest in the medical curriculum (Juškevičienė et al., 2021). Educational agents exert a secondary influence on the learning outcomes of engineering technology students, while their impact on natural sciences and humanities and social sciences students is relatively minor. This conclusion contradicts the view that educational agents can effectively enhance teaching outcomes in natural sciences such as physics and chemistry (Ludvigsen and Arnseth, 2017). This discrepancy arises because natural sciences emphasise students’ perception of objective phenomena through hands-on practice. Educational agents, however, operate solely with virtual objects, depriving students of authentic tactile and olfactory stimuli and diminishing realism. Furthermore, virtual operations primarily rely on keyboard-mouse or touchscreen interfaces, which do not fully replicate genuine practical actions.
4) In terms of sample size, different sample sizes exert a positive influence on students’ learning outcomes. Among these, educational agents exert the greatest impact on small-scale classes (1–50), followed by medium-scale classes (50–100), with the relatively smallest effect observed in large-scale classes (>100). Consistent with Taşdemir’s (2022) findings, this may stem from smaller classes being better suited for writing instruction and peer learning, where collaborative learning environments effectively enhance student outcomes (Ruengtam, 2018). Conversely, in large-scale settings, teaching resources become diluted, reducing opportunities for teachers to encourage student reflection, expression, and communication (Abalde, 2014).
5) In terms of academic level, educational agents exert a positive influence on students’ learning outcomes across all levels. Among these, the enhancement of learning outcomes for university students is most pronounced, followed by high school students, while the impact on primary school students is relatively minimal. Consistent with findings by Kazu and Kurtoglu Yalcin (2021), this may stem from university students’ established study habits, clear learning objectives, and strong self-directed learning capabilities. Their practical application of the educational agent’s knowledge stimulates sustained identification (Robinson et at., 2022). In contrast, high school is a critical period for the formation and development of self-identity, with relatively fixed learning habits, making it less conducive to significant learning outcomes (Du et al., 2020). For primary school students, whose knowledge base is relatively simple and subject-specific reserves limited, educational agents may prove insufficient to capture their learning interest (Scheerens, 2016).

These non-significant results highlight that educational agents’ effectiveness, especially for complex cognitive and non-cognitive outcomes. Study design (sample size, duration), implementation (fidelity, curriculum integration), and measurement consistency play equally important roles as agent characteristics. For example, when agents are used with sufficient fidelity (e.g., integrated into core curriculum, adaptive scaffolding) and in well-powered studies, they have shown significant effects on problem-solving (e.g., Ait Baha et al., 2024) and engagement (e.g., Wang et al., 2023). Thus, non-significant findings should not be interpreted as evidence of agents’ inadequacy but as a call for more rigorous study designs and intentional implementation. Moderator results are similarly theoretically grounded. Chatbots exhibit the strongest effect because they effectively activate all three core mechanisms—personalisation (Cognitive Load Theory), social presence (Social Learning Theory), and perceived ease of use (TAM)—more so than intelligent learning systems or gamified agents. University students benefit most due to higher perceived usefulness and ease of use (TAM), which amplify the impact of agent features on psychological mechanisms. Small-class settings enhance agent-student interaction, strengthening social presence (Social Learning Theory) and personalisation (Cognitive Load Theory), thus driving stronger outcomes.

6) Unlike prior works that focus on narrow tool subsets or undifferentiated AI tools this study explicitly defines and compares three core educational agent subtypes—intelligent learning systems, chatbots, and gamified learning agents. Wu and Yu’s (2024) meta-analysis, for example, focuses solely on AI chatbots, while Hwang (2022) aggregates all AI tools into a single category. This study moves beyond aggregate outcome measures to unpack learning outcomes into nine granular subdimensions (five cognitive, four non-cognitive), revealing nuanced effects that prior meta-analyses miss. Existing works such as Ma and Zhong (2025) focus on broad learning gains while Hwang (2022) limits analysis to academic performance. (c) This study integrates four core learning theories (cognitive load theory, social learning theory, technology acceptance model, self-determination theory) to explain why effects vary, and identifies context-specific effectiveness thresholds—filling the black box of prior descriptive meta-analyses. Wu and Yu (2024) and Hwang (2022) report effect sizes but offer no theoretical explanation for heterogeneity or practical guidance on implementation.
7) This study identified extreme heterogeneity across the 52 included studies (*I*^2^ = 94.51%, *Q* = 930.50, *p* < 0.001), indicating that 94.51% of the variation in effect sizes stems from genuine differences between studies, rather than sampling error (Egger et al., 2022). Interpretation of the overall effect size: The random-effects model (*d* = 0.82) accounts for between-study variation, but the extreme heterogeneity means this value is a weighted average of diverse effects—rather than a one-size-fits-all estimate of educational agents’ impact. For example, effect sizes ranged from *d* = 0.25 (learning interest) to *d* = 1.41 (other subjects), reflecting how context-specific factors (e.g., agent type, task complexity, student characteristics) shape outcomes. Thus, the overall large positive effect should be interpreted as a general trend, not a uniform result across all scenarios. Our moderator analyses (agent type, sample size, etc.) explained some heterogeneity but not all. For instance, while chatbots (*d* = 0.94) outperformed intelligent learning systems (*d* = 0.41), the between-group effect was non-significant (*Chi*^2^ = 1.97, *p* = 0.37), suggesting unmeasured variables (e.g., chatbot interaction depth, integration with curriculum) may further explain variation. This does not invalidate our moderator findings but highlights their conditional nature—effects are stronger under specific contextual conditions. High heterogeneity implies conclusions may not generalize to underrepresented contexts (e.g., low-resource schools, non-Western cultural settings with limited AI adoption). However, our sensitivity test (stable effect sizes after removing any single study: 95% *CI* = 0.54–1.11 for random-effects model) confirms the core finding (educational agents exert a large positive effect) is robust to outliers, mitigating concerns about extreme cases skewing results. Notably, high heterogeneity is common in meta-analyses of educational technology (Wu and Yu, 2024; Zheng et al., 2023), as interventions vary widely in design, implementation, and context. Our study’s value lies in identifying this heterogeneity and laying the groundwork for future research to unpack its sources.

High heterogeneity of this magnitude is expected and typical in meta-analyses of educational technology interventions. Similar levels of heterogeneity have been reported in key prior syntheses, such as Wu and Yu (2024)‘s meta-analysis of AI chatbots in education (*I*^2^ = 92.3%) and Zheng et al. (2023)’ examination of artificial intelligence in learning (*I*^2^ = 93.7%). This reflects the inherent variability in educational contexts, including differences in intervention design, implementation fidelity, student characteristics, and outcome measurement—factors that are unavoidable when synthesising diverse empirical studies in this field. Rather than seeking a single universal effect size for educational agents, this meta-analysis focuses on identifying systematic patterns of effectiveness across contexts. Its goal is explanatory generalisation—understanding how and why educational agents’ impact varies by agent type, academic level, subject, and outcome dimension—rather than predictive generalisation, which would involve applying a fixed effect to all settings. This approach aligns with the core purpose of meta-analyses in educational technology, where unpacking heterogeneity to reveal context-specific patterns is more methodologically meaningful and practically useful than pursuing an overly simplistic universal effect.

Targeted subgroup analyses: explore subgroups based on agent design features (e.g., interaction frequency, feedback type), intervention implementation, and cultural context. For example, subgroup analyses of feedback type could clarify whether motivational feedback (e.g., encouraging messages from gamified agents) drives larger non-cognitive effects than corrective feedback. Analyze subgroups by student characteristics (e.g., special educational needs, digital literacy level) to determine if educational agents’ effectiveness varies by learner background—an unaddressed source of heterogeneity in our study.

Meta-regression analyses: test continuous variables as drivers of effect size variation, including publication year, intervention duration, and agent complexity. Meta-regression could quantify how much each variable explains heterogeneity (e.g., whether newer generative AI agents have larger effect sizes than earlier versions). Use multiple meta-regression to test interactions between variables (e.g., agent type × subject), which may reveal synergistic effects (e.g., chatbots are most effective in language learning, while intelligent learning systems perform better in engineering) that single moderator analyses miss.

8) Educational agents are less effective when tasks require embodied manipulation or direct sensory feedback. The marginally non-significant effect on spatial ability reflects this constraint, as spatial reasoning often depends on physical interaction with three-dimensional objects—something virtual agents cannot fully replicate. Similarly, natural science tasks involving hands-on experimentation (such as chemical reactions or biological dissections) rely on tactile and olfactory feedback that digital agents cannot provide, explaining the relatively smaller effect size for natural sciences compared to engineering or medical subjects. Learner attributes also shape the effectiveness of educational agents. Agents are less impactful for learners with low digital literacy or limited self-regulation. University students benefited most from agents partly due to their advanced digital skills and ability to engage independently with technology. In contrast, primary and high school students—who may lack self-regulation to navigate agent-driven learning without guidance—showed weaker effects. This aligns with prior research indicating that agent effectiveness is contingent on learners’ capacity to utilise adaptive features proactively. Implementation quality is a critical constraint. Short-term interventions (4 weeks or less) and poor curriculum integration limit agent impact. The marginally non-significant trend for problem-solving skills partly stems from interventions that treated agents as supplementary tools rather than integrating them into core learning activities. Sustained, curriculum-aligned use is necessary to activate key psychological mechanisms such as competence support (Self-Determination Theory) and reduced cognitive load (Cognitive Load Theory). Crucially, educational agents are complements to, not replacements for, effective pedagogy. Their strengths lie in personalisation, feedback, and scalability—areas that augment teacher practice—but they cannot replicate the emotional support, contextual judgment, or adaptive scaffolding that human educators provide. The most impactful implementations combine agent-driven support with teacher-led instruction, leveraging the unique strengths of both human and machine intelligence. These boundary conditions highlight that agent effectiveness is context-dependent. Educators should avoid deploying agents for tasks requiring embodied interaction, with learners lacking digital literacy, or in poorly integrated short-term interventions. Instead, agents should be used strategically to enhance, rather than replace, evidence-based pedagogical practices.
9) To translate the study’s findings into actionable practice, this subsection outlines targeted implications for educational designers, institutions, and policymakers, with a focus on pedagogical alignment and evidence-based implementation.

Designers should prioritise adaptive feedback and scaffolding over mere automation. The study’s findings highlight that agents excelling in personalisation (reducing cognitive load) and social presence (fostering relatedness) drive the strongest learning outcomes. For cognitive skills such as creative thinking and academic performance, designers should integrate adaptive feedback that targets individual gaps rather than providing generic responses. For non-cognitive outcomes like motivation, agents should incorporate social presence features such as role enactment (e.g., virtual mentors) to activate observational learning mechanisms. Additionally, designers should avoid overcomplicating interfaces, as ease of use (consistent with the Technology Acceptance Model) enhances effectiveness across learner groups. For tasks requiring spatial ability or embodied interaction, designers should complement agents with physical tools to address the boundary condition of limited sensory feedback.

Institutions should adopt a phased approach to agent implementation, starting with small-scale pilots in contexts where effectiveness is most robust. The study identifies university settings, small classes, and engineering or medical subjects as high-potential contexts for initial deployment. Institutions must invest in teacher training to ensure agents are integrated into curriculum rather than used as supplementary add-ons. Training should focus on aligning agent functions with pedagogical goals—for example, using chatbots to support communication practice in language courses or intelligent learning systems to scaffold problem-solving in engineering. Institutions should also assess learner digital literacy and self-regulation before widespread deployment, providing targeted support for students who may struggle to engage independently with agent-driven learning.

Policymakers and practitioners must avoid overreliance on generative AI agents without clear pedagogical alignment. The study’s marginally non-significant results for problem-solving and engagement highlight that agents without structured scaffolding or curriculum integration fail to deliver meaningful outcomes. Generative AI should not replace teacher-led instruction but rather augment it—for instance, handling routine feedback to free teachers for high-impact tasks such as emotional support and contextualised guidance. Policymakers should encourage standards for pedagogical alignment in educational agent design, ensuring tools are developed with explicit links to learning objectives rather than focusing solely on technical capability. Institutions should monitor agent use to prevent dependency, ensuring students develop independent thinking skills alongside technology literacy.

## 5 Conclusion

This study employs a meta-analysis methodology to investigate the impact of educational agents on student learning outcomes (cognitive and non-cognitive dimensions), drawing upon empirical research literature from internationally authoritative databases spanning 2015 to 2025. It further analyses variations in this impact through four moderating variables: types of agents, sample size, academic level, and subjects. Quantitative synthesis findings indicate that educational agents exert a significant positive effect on student learning outcomes. This conclusion aligns with results from other meta-analyses within the educational domain (Hwang, 2022; Ma and Zhong, 2025; Wu and Yu, 2024). This study reveals that educational agents’ effectiveness varies by outcome and context. While they significantly enhance creative thinking, academic performance, learning motivation, and attitude, non-significant effects on spatial ability, problem-solving, engagement, and interest stem from a combination of study design limitations (small samples, short durations), low intervention fidelity, and measurement inconsistencies—rather than the agents’ inherent flaws. Future research should prioritize larger sample sizes, longer-term implementations, and high-fidelity integration with curricula to fully unlock agents’ potential for complex cognitive and non-cognitive development. This study highlights current issues and challenges in applying educational agents within teaching and learning processes, thereby laying the groundwork for their broader implementation in educational settings. This study strengthens the theoretical foundation of educational agent research by linking empirical findings to four core theories of learning and AI in education. Cognitive load theory explains why agents boost creative thinking and academic performance (by reducing extraneous load), while social learning theory clarifies their impact on non-cognitive outcomes (via social presence). The technology acceptance model and self-determination theory further unpack moderator effects and non-significant findings, respectively. Collectively, these links reveal that educational agents’ effectiveness depends on their ability to optimize cognitive load, foster social presence, and satisfy basic psychological needs. This study delivers distinct novel insights that advance beyond existing meta-analyses in AI and educational technology. Unlike prior works limited to narrow tool subsets, aggregate outcomes, or descriptive findings, we show that educational agents’ effectiveness depends on a synergy of agent type, granular outcome, and context.

This study also has certain limitations: Only 52 pieces of literature met the meta-analysis criteria, thus caution should be exercised when generalising the findings. Future research should broaden data sources to obtain a larger sample size, thereby gaining a more comprehensive understanding of the impact of educational agents on student learning outcomes. Furthermore, this study analysed the effects of only four moderating variables; subsequent research should consider incorporating other potential moderating variables that may influence student learning outcomes. While this study confirms educational agents’ large positive effect on learning outcomes (*d* = 0.82), the very high heterogeneity (*I*^2^ = 94.51%) highlights that this effect is context-dependent. Future research must move beyond broad moderators (e.g., agent type, academic level) to unpack unmeasured sources of variation via targeted subgroup analyses and meta-regression. By addressing these gaps, the field can develop more precise guidelines for designing and implementing educational agents, ensuring their effectiveness is maximized across diverse contexts, learners, and tasks. Finally, the analytical framework of this study warrants further refinement, with classifications requiring greater granularity. Subsequent research should undertake more in-depth meta-analytic quantitative studies, incorporating the growing body of research on the impact of educational agents on student learning outcomes, to derive more comprehensive and objective scientific conclusions.

## Data Availability

The original contributions presented in the study are included in the article/Supplementary material, further inquiries can be directed to the corresponding author.
