# Supplementary material for: Impact of educational agents on student’s learning outcomes: a meta-analysis
Source: Front Psychol. 2026 Feb 24;17:1707196. doi: 10.3389/fpsyg.2026.1707196 (PMC12973282; doi:10.3389/fpsyg.2026.1707196)
Supplement: Supplementary file 1 [file Table_1.docx]

Supplementary Material

# Supplementary Data (A)

| **Author (Year)** | **Sample size** | **Academic level** | **Subjects** | **Types of agents** |
| --- | --- | --- | --- | --- |
| Song et al. (2025) | E36  C36 | Universities | Natural Sciences | Chatbots |
| Liu et al. (2025) | E46  C47 | Primary Schools | Natural Sciences | Gamified Learning Agents |
| Tajik (2025) | E23  C22 | Universities | Natural Sciences | Intelligent Learning Systems |
| Teimourtash (2025) | E20  C20 | Universities | Natural Sciences | Chatbots |
| Tai and Chen (2024) | E31  C31 | Primary Schools | Humanities and Social Sciences | Chatbots |
| Liu et al. (2024) | E47  C48 | Primary Schools | Humanities and Social Sciences | Chatbots |
| Ait Baha et al. (2024) | E34  C38 | High Schools | Humanities and Social Sciences | Chatbots |
| Ortega-Ochoa et al. (2024) | E101  C95 | Universities | Humanities and Social Sciences | Chatbots |
| Liang et al. (2024) | E17  C22 | Primary Schools | Humanities and Social Sciences | Chatbots |
| Chen et al. (2024) | E20  C21 | Universities | Natural Sciences | Chatbots |
| Hwang and Zhang (2024) | E30  C26 | High Schools | Natural Sciences | Gamified Learning Agents |
| Zheng (2024) | E42  C42 | High Schools | Humanities and Social Sciences | Chatbots |
| Bahari et al. (2024) | E137  C137 | Universities | Humanities and Social Sciences | Chatbots |
| Liu et al. (2023) | E23  C21 | High Schools | Humanities and Social Sciences | Chatbots |
| Nicolaidou et al. (2023) | E20  C20 | Universities | Humanities and Social Sciences | Intelligent Learning Systems |
| Liu and Chen (2023) | E18  C18 | Primary Schools | Natural Sciences | Chatbots |
| Qasem et al. (2023) | E20  C20 | Universities | Natural Sciences | Chatbots |
| Shi et al. (2023) | E156  C156 | Universities | Natural Sciences | Intelligent Learning Systems |
| Wang et al. (2023) | E87  C68 | High Schools | Natural Sciences | Intelligent Learning Systems |
| M.-H. Hsu et al. (2023) | E24  C23 | Universities | Humanities and Social Sciences | Chatbots |
| Yuan et al. (2023) | E20  C20 | Universities | Engineering Technology | Chatbots |
| H.-L. Hsu et al. (2023) | E24  C26 | Universities | Humanities and Social Sciences | Chatbots |
| Yilmaz and Yilmaz (2023) | E21  C24 | Universities | Natural Sciences | Chatbots |
| Huang et al. (2023) | E43  C59 | Universities | Natural Sciences | Intelligent Learning Systems |
| Tai (2022) | E35  C30 | Universities | Humanities and Social Sciences | Chatbots |
| Chien et al. (2022) | E37  C36 | High Schools | Natural Sciences | Chatbots |
| Lee et al. (2022) | E18  C20 | Universities | Natural Sciences | Chatbots |
| Hwang et al. (2022) | E23  C20 | Primary Schools | Humanities and Social Sciences | Intelligent Learning Systems |
| Han et al. (2022) | E30  C31 | Universities | Others | Chatbots |
| Liu et al. (2022) | E45  C23 | Primary Schools | Humanities and Social Sciences | Chatbots |
| Fidan and Gencel (2022) | E54  C40 | Universities | Others | Chatbots |
| Song and Kim (2021) | E27  C29 | Universities | Others | Chatbots |
| Vázquez-Cano et al. (2021) | E52  C51 | Universities | Humanities and Social Sciences | Chatbots |
| Klos et al. (2021) | E39  C34 | Universities | Others | Chatbots |
| Donnermann et al. (2021) | E20  C21 | Universities | Natural Sciences | Chatbots |
| Yin et al. (2021) | E51  C48 | Universities | Natural Sciences | Chatbots |
| Lin et al. (2020) | E31  C24 | Universities | Natural Sciences | Gamified Learning Agents |
| Chen et al. (2020) | E19  C39 | Universities | Humanities and Social Sciences | Chatbots |
| Dizon (2020) | E13  C15 | Universities | Humanities and Social Sciences | Chatbots |
| Winkler et al. (2020) | E37  C35 | Universities | Natural Sciences | Chatbots |
| Abbasi et al. (2019) | E55  C55 | Universities | Natural Sciences | Chatbots |
| Nghi et al. (2019) | E100  C100 | Universities | Humanities and Social Sciences | Chatbots |
| Fidan and Tuncel (2019) | E30  C30 | High Schools | Natural Sciences | Intelligent Learning Systems |
| Hsieh and Chen (2019) | E67  C70 | High Schools | Natural Sciences | Intelligent Learning Systems |
| Pellas and Vosinakis (2018) | E25  C25 | High Schools | Natural Sciences | Gamified Learning Agents |
| Tegos and Demetriadis (2017) | E38  C34 | Universities | Natural Sciences | Chatbots |
| Madathil et al. (2017) | E83  C82 | Universities | Natural Sciences | Intelligent Learning Systems |
| Calvo‐Ferrer (2017) | E32  C27 | Universities | Humanities and Social Sciences | Gamified Learning Agents |
| Tegos et al. (2016) | E32  C32 | Universities | Natural Sciences | Chatbots |
| Julià and Antolí (2016) | E9  C12 | Primary Schools | Engineering Technology | Chatbots |
| Tegos et al. (2015) | E21  C22 | Universities | Natural Sciences | Chatbots |
| Zafar and Albidewi (2015) | E29  C28 | Universities | Natural Sciences | Intelligent Learning Systems |

# Supplementary Data (B)

| **Author (Year)** | **E** | **M** | **SD** | **C** | **M** | **SD** |
| --- | --- | --- | --- | --- | --- | --- |
| Song et al. (2025) | 36 | 3.64 | 0.53 | 36 | 3.14 | 0.66 |
| Liu et al. (2025) | 46 | 4.03 | 1.02 | 47 | 3.12 | 1.27 |
| Tajik (2025) | 23 | 4.16 | 0.81 | 22 | 3.58 | 0.61 |
| Teimourtash (2025) | 20 | 44.30 | 3.34 | 20 | 36.55 | 2.32 |
| Tai and Chen (2024) | 31 | 91.58 | 22.47 | 31 | 81.35 | 25.25 |
| Liu et al. (2024) | 47 | 3.81 | 0.75 | 48 | 3.15 | 0.78 |
| Ait Baha et al. (2024) | 34 | 10.97 | 4.03 | 38 | 10.50 | 3.34 |
| Ortega-Ochoa et al. (2024) | 101 | 3.23 | 1.22 | 95 | 4.04 | 0.87 |
| Liang et al. (2024) | 17 | 4.40 | 0.57 | 22 | 3.66 | 0.90 |
| Chen et al. (2024) | 20 | 11.90 | 2.09 | 21 | 10.79 | 1.02 |
| Hwang and Zhang (2024) | 30 | 61.87 | 3.25 | 26 | 52.01 | 3.39 |
| Zheng (2024) | 42 | 47.09 | 14.56 | 42 | 48.23 | 18.06 |
| Bahari et al. (2024) | 137 | 70.83 | 4.36 | 137 | 47.93 | 4.81 |
| Liu et al. (2023) | 23 | 80.96 | 16.16 | 21 | 68.46 | 19.78 |
| Nicolaidou et al. (2023) | 20 | 3.78 | 2.25 | 20 | 4.66 | 2.45 |
| Liu and Chen (2023) | 18 | 78.76 | 16.92 | 18 | 55.33 | 24.49 |
| Qasem et al. (2023) | 20 | 14.85 | 3.63 | 20 | 11.70 | 3.21 |
| Shi et al. (2023) | 156 | 73.82 | 11.68 | 156 | 70.38 | 9.80 |
| Wang et al. (2023) | 87 | 65.52 | 19.24 | 68 | 60.85 | 17.89 |
| M.-H. Hsu et al. (2023) | 24 | 141.25 | 17.46 | 24 | 73.21 | 16.10 |
| Yuan et al. (2023) | 20 | 79.50 | 12.34 | 20 | 50.50 | 10.50 |
| H.-L. Hsu et al. (2023) | 24 | 96.78 | 18.65 | 26 | 90.38 | 18.54 |
| Yilmaz and Yilmaz (2023) | 21 | 126.73 | 8.34 | 24 | 112.61 | 15.32 |
| Huang et al. (2023) | 43 | 55.00 | 19.76 | 59 | 50.69 | 23.00 |
| Tai (2022) | 35 | 85.03 | 5.24 | 30 | 79.40 | 5.22 |
| Hwang et al. (2022) | 23 | 70.78 | 17.86 | 20 | 57.60 | 18.06 |
| Chien et al. (2022) | 37 | 64.70 | 16.66 | 36 | 64.83 | 17.51 |
| Lee et al. (2022) | 18 | 4.36 | 0.59 | 20 | 3.67 | 0.47 |
| Han et al. (2022) | 30 | 10.07 | 2.24 | 31 | 10.77 | 1.70 |
| Liu et al. (2022) | 45 | 3.76 | 0.96 | 23 | 3.60 | 1.02 |
| Fidan and Gencel (2022) | 54 | 84.81 | 1.23 | 40 | 75.50 | 1.48 |
| Song and Kim (2021) | 27 | 63.87 | 22.68 | 29 | 53.00 | 20.10 |
| Vázquez-Cano et al. (2021) | 52 | 32.13 | 9.53 | 51 | 28.47 | 9.43 |
| Klos et al. (2021) | 39 | 13.04 | 7.12 | 34 | 16.26 | 5.79 |
| Donnermann et al. (2021) | 20 | 5.33 | 0.64 | 21 | 5.34 | 0.57 |
| Yin et al. (2021) | 51 | 5.82 | 1.75 | 48 | 5.75 | 2.27 |
| Lin et al. (2020) | 31 | 6.55 | 2.24 | 24 | 8.55 | 3.97 |
| Chen et al. (2020) | 19 | 91.33 | 11.77 | 39 | 88.03 | 12.35 |
| Dizon (2020) | 13 | 6.69 | 1.93 | 15 | 6.20 | 1.66 |
| Winkler et al. (2020) | 37 | 65.40 | 27.10 | 35 | 44.00 | 21.90 |
| Abbasi et al. (2019) | 55 | 57.20 | 9.86 | 55 | 40.62 | 9.47 |
| Nghi et al. (2019) | 100 | 6.60 | 1.63 | 100 | 6.60 | 1.63 |
| Fidan and Tuncel (2019) | 30 | 29.23 | 4.56 | 30 | 22.73 | 5.42 |
| Hsieh and Chen (2019) | 67 | 3.31 | 1.07 | 70 | 4.08 | 0.92 |
| Pellas and Vosinakis (2018) | 25 | 4.34 | 1.27 | 25 | 3.81 | 1.17 |
| Tegos and Demetriadis (2017) | 38 | 12.12 | 4.41 | 34 | 9.45 | 3.76 |
| Madathil et al. (2017) | 83 | 6.08 | 5.30 | 82 | 5.69 | 1.45 |
| Calvo‐Ferrer (2017) | 32 | 35.56 | 6.49 | 27 | 31.63 | 7.04 |
| Tegos et al. (2016) | 32 | 14.01 | 3.68 | 32 | 10.13 | 4.48 |
| Julià and Antolí (2016) | 9 | 58.40 | 8.80 | 12 | 57.30 | 10.20 |
| Tegos et al. (2015) | 21 | 7.92 | 1.50 | 22 | 6.59 | 1.29 |
| Zafar and Albidewi (2015) | 29 | 20.52 | 1.73 | 28 | 17.68 | 1.60 |

Abbasi, S., Kazi, H., & Hussaini, N. N. (2019). Effect of chatbot systems on students learning outcomes. *Sylwan, 163*(10), 49-63.

Ait Baha, T., El Hajji, M., Es-Saady, Y., & Fadili, H. (2024). The impact of educational chatbot on student learning experience. *Education and Information Technologies, 29*(8), 10153-10176.

Bahari, A., Smith, M., & Scott, H. (2024). Examining the Impact of Chatbot-Based Language Learning Support, Adaptive Learning Algorithms, and Virtual Reality Language Immersion on EFL Learners’ Language Learning Proficiency and Self-Regulated Learning Skills.

Calvo‐Ferrer, J. R. (2017). Educational games as stand‐alone learning tools and their motivational effect on L 2 vocabulary acquisition and perceived learning gains. *British journal of educational technology, 48*(2), 264-278.

Chen, A., Wei, Y., Le, H., & Zhang, Y. (2024). Learning by teaching with ChatGPT: The effect of teachable ChatGPT agent on programming education. *British Journal of Educational Technology*.

Chen, H.-L., Vicki Widarso, G., & Sutrisno, H. (2020). A chatbot for learning Chinese: Learning achievement and technology acceptance. *Journal of Educational Computing Research, 58*(6), 1161-1189.

Chien, Y.-C., Wu, T.-T., Lai, C.-H., & Huang, Y.-M. (2022). Investigation of the influence of artificial intelligence markup language-based LINE ChatBot in contextual English learning. *Frontiers in Psychology, 13*, 785752.

Dizon, G. (2020). Evaluating intelligent personal assistants for L2 listening and speaking development.

Donnermann, M., Lein, M., Messingschlager, T., Riedmann, A., Schaper, P., Steinhaeusser, S., & Lugrin, B. (2021). Social robots and gamification for technology supported learning: An empirical study on engagement and motivation. *Computers in Human Behavior, 121*, 106792.

Fidan, M., & Gencel, N. (2022). Supporting the instructional videos with chatbot and peer feedback mechanisms in online learning: The effects on learning performance and intrinsic motivation. *Journal of Educational Computing Research, 60*(7), 1716-1741.

Fidan, M., & Tuncel, M. (2019). Integrating augmented reality into problem based learning: The effects on learning achievement and attitude in physics education. *Computers & Education, 142*, 103635.

Han, J.-W., Park, J., & Lee, H. (2022). Analysis of the effect of an artificial intelligence chatbot educational program on non-face-to-face classes: a quasi-experimental study. *BMC Medical Education, 22*(1), 830.

Hsieh, M.-C., & Chen, S.-H. (2019). Intelligence augmented reality tutoring system for mathematics teaching and learning. *Journal of Internet Technology, 20*(5), 1673-1681.

Hsu, H.-L., Chen, H. H.-J., & Todd, A. G. (2023). Investigating the impact of the Amazon Alexa on the development of L2 listening and speaking skills. *Interactive Learning Environments, 31*(9), 5732-5745.

Hsu, M.-H., Chen, P.-S., & Yu, C.-S. (2023). Proposing a task-oriented chatbot system for EFL learners speaking practice. *Interactive Learning Environments, 31*(7), 4297-4308.

Huang, A. Y., Lu, O. H., & Yang, S. J. (2023). Effects of artificial Intelligence–Enabled personalized recommendations on learners’ learning engagement, motivation, and outcomes in a flipped classroom. *Computers & Education, 194*, 104684.

Hwang, G.-J., & Zhang, D. (2024). Effects of an adaptive computer agent-based digital game on EFL students’ English learning outcomes. *Educational technology research and development, 72*(6), 3271-3294.

Hwang, W.-Y., Guo, B.-C., Hoang, A., Chang, C.-C., & Wu, N.-T. (2022). Facilitating authentic contextual EFL speaking and conversation with smart mechanisms and investigating its influence on learning achievements. *Computer Assisted Language Learning*, 1-27.

Julià, C., & Antolí, J. Ò. (2016). Spatial ability learning through educational robotics. *International Journal of Technology and Design Education, 26*, 185-203.

Klos, M. C., Escoredo, M., Joerin, A., Lemos, V. N., Rauws, M., & Bunge, E. L. (2021). Artificial intelligence–based chatbot for anxiety and depression in university students: pilot randomized controlled trial. *JMIR formative research, 5*(8), e20678.

Lee, Y.-F., Hwang, G.-J., & Chen, P.-Y. (2022). Impacts of an AI-based cha bot on college students’ after-class review, academic performance, self-efficacy, learning attitude, and motivation. *Educational technology research and development, 70*(5), 1843-1865.

Liang, H. Y., Hwang, G. J., Hsu, T. Y., & Yeh, J. Y. (2024). Effect of an AI‐based chatbot on students' learning performance in alternate reality game‐based museum learning. *British journal of educational technology*.

Lin, C.-J., Hwang, G.-J., Fu, Q.-K., & Cao, Y.-H. (2020). Facilitating EFL students’ English grammar learning performance and behaviors: A contextual gaming approach. *Computers & Education, 152*, 103876.

Liu, C.-C., Chen, W.-J., Lo, F.-y., Chang, C.-H., & Lin, H.-M. (2024). Teachable Q&A Agent: The Effect of Chatbot Training by Students on Reading Interest and Engagement. *Journal of Educational Computing Research, 62*(4), 1122-1154.

Liu, C.-C., Liao, M.-G., Chang, C.-H., & Lin, H.-M. (2022). An analysis of children’interaction with an AI chatbot and its impact on their interest in reading. *Computers & Education, 189*, 104576.

Liu, C.-C., Lin, Y.-Y., Lo, F.-y., Chang, C.-H., & Lin, H.-M. (2025). From readers to players: Exploring student engagement in a gamified metaverse and its effect on reading interest. *Education and Information Technologies, 30*(1), 421-447.

Liu, P.-L., & Chen, C.-J. (2023). Using an AI-based object detection translation application for English vocabulary learning. *Educational Technology & Society, 26*(3), 5-20.

Liu, Y.-F., Hwang, W.-Y., & Su, C.-H. (2023). Investigating the impact of context-awareness smart learning mechanism on EFL conversation learning. *Interactive Learning Environments*, 1-16.

Madathil, K. C., Frady, K., Hartley, R., Bertrand, J., Alfred, M., & Gramopadhye, A. (2017). An empirical study investigating the effectiveness of integrating virtual reality-based case studies into an online asynchronous learning environment. *Computers in Education Journal, 8*(3).

Nghi, T. T., Phuc, T. H., & Thang, N. T. (2019). Applying AI chatbot for teaching a foreign language: An empirical research. *International Journal of Scientific and Technology Research, 8*(12), 897-902.

Nicolaidou, I., Pissas, P., & Boglou, D. (2023). Comparing immersive virtual reality to mobile applications in foreign language learning in higher education: A quasi-experiment. *Interactive Learning Environments, 31*(4), 2001-2015.

Pellas, N., & Vosinakis, S. (2018). The effect of simulation games on learning computer programming: A comparative study on high school students’ learning performance by assessing computational problem-solving strategies. *Education and Information Technologies, 23*(6), 2423-2452.

Qasem, F., Ghaleb, M., Mahdi, H. S., Al Khateeb, A., & Al Fadda, H. (2023). Dialog chatbot as an interactive online tool in enhancing ESP vocabulary learning. *Saudi Journal of Language Studies, 3*(2), 76-86.

Shi, L., Muhammad Umer, A., & Shi, Y. (2023). Utilizing AI models to optimize blended teaching effectiveness in college-level English education. *Cogent Education, 10*(2), 2282804.

Song, D., & Kim, D. (2021). Effects of self-regulation scaffolding on online participation and learning outcomes. *Journal of Research on Technology in Education, 53*(3), 249-263.

Song, Y., Huang, L., Zheng, L., Fan, M., & Liu, Z. (2025). Interactions with generative AI chatbots: unveiling dialogic dynamics, students’ perceptions, and practical competencies in creative problem-solving. *International Journal of Educational Technology in Higher Education, 22*(1), 12.

Tai, T.-Y. (2022). Effects of intelligent personal assistants on EFL learners’ oral proficiency outside the classroom. *Computer Assisted Language Learning*, 1-30.

Tai, T.-Y., & Chen, H. H.-J. (2024). The impact of intelligent personal assistants on adolescent EFL learners’ listening comprehension. *Computer Assisted Language Learning, 37*(3), 433-460.

Tajik, A. (2025). Exploring the role of AI-driven dynamic writing platforms in improving EFL learners' writing skills and fostering their motivation.

Tegos, S., & Demetriadis, S. (2017). Conversational agents improve peer learning through building on prior knowledge. *Journal of Educational Technology & Society, 20*(1), 99-111.

Tegos, S., Demetriadis, S., & Karakostas, A. (2015). Promoting academically productive talk with conversational agent interventions in collaborative learning settings. *Computers & Education, 87*, 309-325.

Tegos, S., Demetriadis, S., Papadopoulos, P. M., & Weinberger, A. (2016). Conversational agents for academically productive talk: A comparison of directed and undirected agent interventions. *International Journal of Computer-Supported Collaborative Learning, 11*, 417-440.

Teimourtash, M. Enriching the Zone of Audacity through AI-Assisted Corrective Feedback: Iranian EFL Learners’ Writing Anxiety Revisited. *English Education, 3*(2), 1-21.

Vázquez-Cano, E., Mengual-Andrés, S., & López-Meneses, E. (2021). Chatbot to improve learning punctuation in Spanish and to enhance open and flexible learning environments. *International Journal of Educational Technology in Higher Education, 18*, 1-20.

Wang, S., Christensen, C., Cui, W., Tong, R., Yarnall, L., Shear, L., & Feng, M. (2023). When adaptive learning is effective learning: comparison of an adaptive learning system to teacher-led instruction. *Interactive Learning Environments, 31*(2), 793-803.

Winkler, R., Hobert, S., Salovaara, A., Söllner, M., & Leimeister, J. M. (2020). Sara, the lecturer: Improving learning in online education with a scaffolding-based conversational agent. Proceedings of the 2020 CHI conference on human factors in computing systems,

Yilmaz, R., & Yilmaz, F. G. K. (2023). The effect of generative artificial intelligence (AI)-based tool use on students' computational thinking skills, programming self-efficacy and motivation. *Computers and Education: Artificial Intelligence, 4*, 100147.

Yin, J., Goh, T.-T., Yang, B., & Xiaobin, Y. (2021). Conversation technology with micro-learning: The impact of chatbot-based learning on students’ learning motivation and performance. *Journal of Educational Computing Research, 59*(1), 154-177.

Yuan, C.-C., Li, C.-H., & Peng, C.-C. (2023). Development of mobile interactive courses based on an artificial intelligence chatbot on the communication software LINE. *Interactive Learning Environments, 31*(6), 3562-3576.

Zafar, A., & Albidewi, I. (2015). Evaluation study of eLGuide: A framework for adaptive e‐learning. *Computer Applications in Engineering Education, 23*(4), 542-555.

Zheng, S. (2024). The Effects of Chatbot Use on Foreign Language Reading Anxiety and Reading Performance among Chinese Secondary School Students. *Computers and Education: Artificial Intelligence*, 100271.
